# Bonding Behaviour of Steel Fibres in UHPFRC Based on Alkali-Activated Slag

**DOI:** 10.3390/ma15051930

**Published:** 2022-03-04

**Authors:** Alexander Wetzel, Daniela Göbel, Maximilian Schleiting, Niels Wiemer, Bernhard Middendorf

**Affiliations:** Department of Structural Materials and Construction Chemistry, University of Kassel, 34125 Kassel, Germany; daniela.goebel@uni-kassel.de (D.G.); schleiting@uni-kassel.de (M.S.); niels.wiemer@uni-kassel.de (N.W.); middendorf@uni-kassel.de (B.M.)

**Keywords:** ultra-high-performance concrete, fibre pullout, silica fume, potassium waterglass, fibre reinforcement

## Abstract

The mechanical performance of fibre-reinforced ultra-high-performance concrete based on alkali-activated slag was investigated, concentrating on the use of steel fibres. The flexural strength is slightly higher compared to the UHPC based on Ordinary Portland Cement (OPC) as the binder. Correlating the flexural strength test with multiple fibre-pullout tests, an increase in the bonding behaviour at the interfacial-transition zone of the AAM-UHPC was found compared to the OPC-UHPC. Microstructural investigations on the fibres after storage in an artificial pore solution and a potassium waterglass indicated a dissolution of the metallic surface. This occurred more strongly with the potassium waterglass, which was used as an activator solution in the case of the AAM-UHPC. From this, it can be assumed that the stronger bond results from this initial etching for steel fibres in the AAM-UHPC compared to the OPC-UHPC. The difference in the bond strength of both fibre types, the brass-coated steel fibres and the stainless-steel fibres, was rather low for the AAM-UHPC compared to the OPC-UHPC.

## 1. Introduction

The main characteristics of ultra-high-performance concrete are its low capillary porosity and its high compressive strength [1]. Both characteristics evolve from its low water/binder ratio and its microstructure optimization by the use of reactive fines such as silica fumes. An issue with OPC-based ultra-high-performance concrete is the high content of cement. Due to the benefits of its high compressive strength, less concrete is needed and therefore, in terms of the CO_2_-, water-, and material-footprint, this type of concrete can be beneficial [2]. Anyhow, the aim is to lower these footprints further by optimizing the composition. Alkali-activated material is based on alumo-silicatic precursors, most times slags, fly ashes, or metakaolin and an activator, usually alkali-waterglass or alkali-hydroxide [3,4,5]. In prior work, it was reported that it is possible to produce a UHPC without Portland cement by using alkali-activated slag [6]. Furthermore, it was shown that due to the use of silica fumes in certain amounts, the rheological behaviour of the AAM-UHPC could be improved and a water/binder ratio even lower than 0.2 could be realized without using any superplasticizers. Anyhow, the use of superplasticizers in that high-alkaline system is rather inefficient [7].

In the present study, this mix was optimized in terms of setting time and compressive strength. Furthermore, a different kind of silica fume from ferro-silicium production was used to reduce the cost of the final product. The main aspects of this paper, however, are the mechanical characteristics under variations of the added steel fibres. For this reason, flexural-bending tests were conducted as well as fibre-pullout tests [8]. In preliminary investigations on UHPCs based on OPC single- and multiple-fibres, pullout tests were correlated with flexural-bending tests [9]. It is well known that steel fibres improve the post-cracking behaviour of UHPCs [10]. Steel fibres in the size range of about 6–60 mm length and 0.15–0.50 mm in diameter are used for UHPCs in an amount of about 1–3 vol% [11]. Beneath the benefits of higher bending strength and improved post-cracking behaviour, the fibres preserve the concrete of macrocracks due to interlocking, resulting in the uniform distribution of microcracks [12]. Steel fibres show a tensile strength in the range of 1000–2500 MPa. Under load, a crack occurs in the concrete, and when it widens, the fibres are pulled out; due to frictional forces, improved post-cracking behaviour was displayed. In the case of fibre failure, the post-cracking behaviour, which might be described as pseudo-ductile, gets lost. Thus, the geometry of the fibres and the bonding strength between fibres and the inorganic binder matrix are essential [13,14,15]. Therefore, the presented investigations focus on the bond behaviour of the steel fibres in ultra-high-performance concrete based on alkali-activated slag. It is proven whether the post-cracking behaviour is enhanced, as is known for the UHPC based on OPC [9]. The correlation between the flexural strength and the pullout strength is discussed.

## 2. Materials and Methods

The UHPC studied and optimized in these investigations is based on alkali-activated, ground-granulated, blast-furnace slag, hereinafter referred to as slag. The activator solution used was potassium waterglass. In prior work, a mix of alkali-hydroxide and a different type of waterglass was used [6]. Two different potassium waterglass solutions were used here, where the first one had a modulus (SiO_2_:K_2_O; mol%) of 3.9 and the second one of 1.0. Anyhow, the main aspect of the UHPC is its low porosity, which is gained by adding a certain amount of fines following packing-density optimization [6]. Here, beneath quartz powder, two different types of silica fumes were used. The first one was a co-product of metallic-silicium production, and the second one was a co-product of ferro-silicium-alloy production in electric-arc furnaces; the latter is denoted as the f-silica fume. The grain-size distributions and the specific surfaces of all of the precursors are given in Table 1. The specific surfaces of the fines were estimated with Blaine, for the slag and by the gas adsorption after BET for the two types of silica fumes.

The mixes were prepared in intensive mixers either with a volume of 1 dm^3^ or 3 dm^3^. All measurements were performed in an air-conditioned laboratory at 65% relative humidity and at 20 °C. For the mixing procedure, first, the dry components without the sand were mixed for 60 s, followed by the addition of the waterglass solution, followed by further mixing for 180 s. Finally, after a break to remove the residual dry components on the mixing tool and the container, the sand was added, and after another 150 s, the mix was directly poured into the moulds. If fibres were used, they were added directly after the addition of the sand, taking care not to agglomerate the fibres. After covering, the samples were stored at 20 °C and 65% r.h. After one day, the samples were demoulded and stored in closed plastic bags at 20 °C until testing. Beneath the setting time, the spread flow was measured directly after mixing under standard conditions (20 °C/65% r.h.). Flexural strength (DIN EN 12390-5, [16]) and compressive strength (DIN EN 12390-3, [17]) were measured on prisms measuring 40 × 40 × 160 mm^3^. Therefore, after 4-point flexural strength testing, the residual pieces were used for compressive strength tests. 

To compare the results of the flexural strengths with each other, the averaged flexural strengths were compared at three defined deformation values. These were defined in accordance with the DafStb-guideline “Stahlfaserbeton” for deflections of δ_L1_ = 0.5 mm, δ_L2_ = 3.5 mm, and at maximum load [18]. For small deformations, the flexural strength is referred to as the service-load range $(f_{cflk,\;L1}^{f}$), and for large deformations, as the fracture range ($f_{cflk,\;L2}^{f}$). The statistical-characteristic values of the post-cracking flexural strength (N/mm^2^) are determined by the following equations [18]:

(1) $$f_{cflm,\;L1}^{f}=\frac{1}{n}\;\sum_{\;i=1}^{\;n}\frac{M_{0.5i}}{W_{0.5i}}=\frac{1}{n}\;\sum_{\;i=1}^{\;n}\frac{\frac{F_{0.5i\;}}{2}\cdot \frac{l}{3}}{\frac{b_{i}\;\cdot \;h_{i}^{2}}{6}}=\frac{1}{n}\;\sum_{\;i=1}^{\;n}\frac{F_{0.5i\;}\cdot \;l}{b_{i}\;\cdot \;h_{i}^{2}}$$

(2) $$f_{cflm,\;L2}^{f}=\frac{1}{n}\overset{n}{\underset{i=1}{\sum }}\frac{F_{3.5i\;}\cdot l}{b_{i}\;\cdot \;h_{i}^{2}}$$

*F* is the applied load (N), *l* is the support distance (mm), *b* is the section width (mm), *h* is the section height (mm), *M* is the moment (Nmm), *W* is the section modulus (mm^3^), and the index *i* is the sample number.

Due to the post-cracking flexural strengths in the service- and fracture-load range, the performance characteristics of the fibre concretes are clearly describable. The characteristic values are required for the performance factor. These results depend on the coefficient of variation (*v*):

For v > 0.25

(3) $$f_{cflk,Li\;}^{f}=0.51\;\cdot \;f_{cflm,Li\;}^{f}$$

for *v* > 0.25

(4) $$f_{cflk,\;Li}^{f}=f_{cflm,Li\;}^{f}\;\cdot \;\left(1-t\;\cdot v\right)$$

*t* is the threshold for t distribution (5%-fractile).

**Figure 1:**
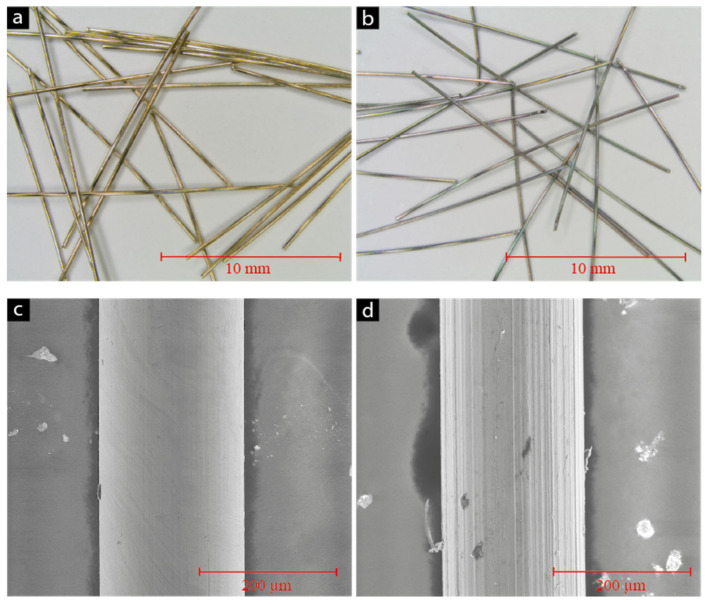
Steel fibres in light microscope (**a**) brass-coated steel fibres (SF), (**b**) stainless-steel fibres (SSF); and in SEM (low vacuum, secondary electron mode) (**c**) brass-coated steel fibre (SF), (**d**) stainless-steel fibre (SSF).

For further investigation on the bond behaviour between the metallic fibre and the matrix, fibre-pullout tests were made. For the flexural strength and the fibre-pullout tests, two different fibres were used (Figure 1); brass-coated steel fibres (SFs), which are commonly used in UHPCs to increase the post-cracking behaviour, on the one hand, and on the other hand, stainless-steel fibres (SSFs). Both fibre types had a length of 17 mm ± 10%. The SF consists of high-strength steel, with a tensile strength of approx. 2000 N/mm^2^ and a thickness of 0.2 mm ± 15%. The SSFs are austenitic chromium–nickel steel fibres with a tensile strength of approx. 1980 N/mm^2^ and a thickness of 0.2 mm ± 10%. The difference in surface roughness is obvious from the SEM pictures (Figure 1c,d).

**Figure 2:**
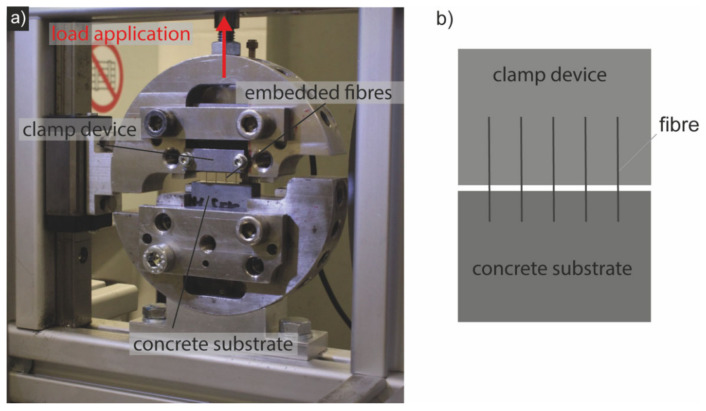
CTS device for the fibre-pullout tests: (**a**) installed sample for multiple-fibre pullout (5 fibres) in the CTS device during a pullout, (**b**) sketch of setup.

Fibre-pullout tests were conducted using a ’Compact-Tension-Shear’ device (CTS test) to quantify the bond between the fibres and the binder matrix [8,9]. For the fibre-pullout tests, the CTS device was used to measure 5 fibres simultaneously (Figure 2). The fibre-pullout test was conducted in displacement control with a constant speed of 0.1 mm/s (using a 150 kN Zwick/Roell testing rig). The fibres were embedded in a gauge, guaranteeing a straight-embedding angle and a defined distance to the sample rim and between each fibre. For further descriptions, see Wiemer et al. 2020 [9], where the bond behaviour of the OPC-UHPC and the same fibre types as in the present work were tested. 

The maximum bond stress (*τ_max_*) is calculated by the following equation:

(5) $$\tau_{max}=\frac{F_{max}/fibre}{d_{f}\;\cdot \;\pi \;\cdot \;l_{e}}$$

where *F_max_* is the maximum pullout load, df is the fibre diameter, and *l_e_* is the embedded fibre length *l_e_*. The maximum fibre stress is a measure for the efficiency of the fibre utilization [13]:

(6) $$\sigma_{f,max}=\frac{F_{max}/fibre}{A_{f}}=\frac{F_{max}/fibre}{\pi \;\cdot \;\frac{{\left(d_{f}\right)}^{2}}{4}}$$

The utilization factor (*u_f_*) quantifies the effectiveness of the fibres in percent. Its value is defined as the ratio of the maximum tensile stress *σ_max_* of the fibre after pulling out the tensile strength *f_y_* of the fibre. A utilization of more than 100% leads to a failure of the fibres.

(7) $$u_{f}=\frac{\sigma_{f,max}}{f_{y}}\;\cdot 100$$

Additionally, force–displacement diagrams are used to quantify dissipated energy or pullout work (*W_P_*). In the present work, the dissipated energy is defined as the integral over the entire embedding length of 5 mm, which indicates the work needed to pull out the fibres. The dissipated energy is given in the unit of work (Nm).

(8) $$W_{P}=\int_{s=0}^{s=5}\frac{F\left(s\right)}{5}ds$$

In terms of the flexural strength, the dissipated energy can be considered as the work that must be applied for the corresponding deflection of a specimen. In this case, the calculation is made using a deflection in the centre of the prism from 0 mm to 5 mm.

After the pullout of the fibres, the interface at the embedding depth was characterized via scanning electron microscopy. The single fibres were measured in a low vacuum (50 Pa) with an acceleration voltage of 15 kV using a large-field detector for the secondary electron (SE) mode and a low-voltage high-contrast detector (vCD) for the backscattered electron (BSE) mode.

## 3. Results

The properties of fresh and hardened concrete were investigated for different types of mixes (Table 2). As reported in [6], the M1 mix was composed of slag and silica fumes as precursors, quartz sand and quartz powder were used as aggregates, and a mix of potassium waterglass and potassium hydroxide was used as the activator solution. In mix M2, a different type of waterglass solution was used (Wöllner K57N), with a solid content of 53% and a modulus (molar ratio of SiO_2_:K_2_O) of 1 after the manufacturer´s specification. Most obviously, the setting time improved to more than 100 min compared to M1, the compressive strength was increased, and the workability was increased, showing a slump flow of 340 mm, although the water/binder ratio was reduced. A reduction in the w/b ratio (M3) led to a further increase in compressive strength. Beneath the slump flow, the performance of M4 was comparable to M3; the difference in the mix design was a different type of silica fume (SF2), which was derived from ferro-silicium production. Further measurements with fibre reinforcement were conducted using the formulation M2 to counteract the workability decrease using fibres.

**Figure 3:**
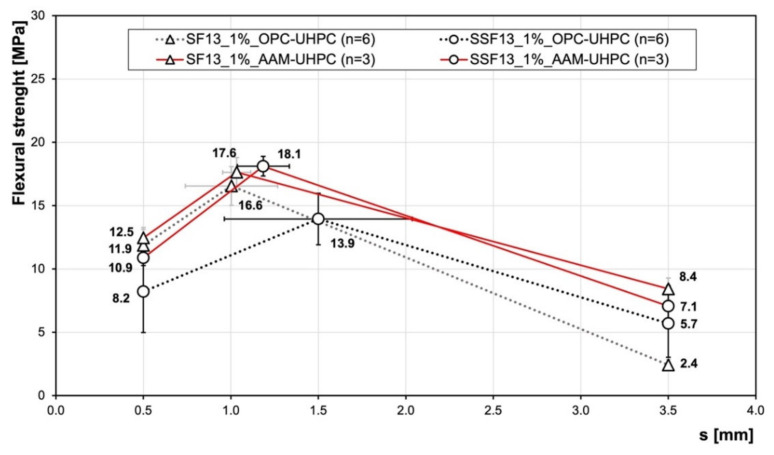
Flexural strength of AAM-UHPC compared to OPC-UHPC [9] after 7 days. The number n of tests is given in the legend.

**Figure 4:**
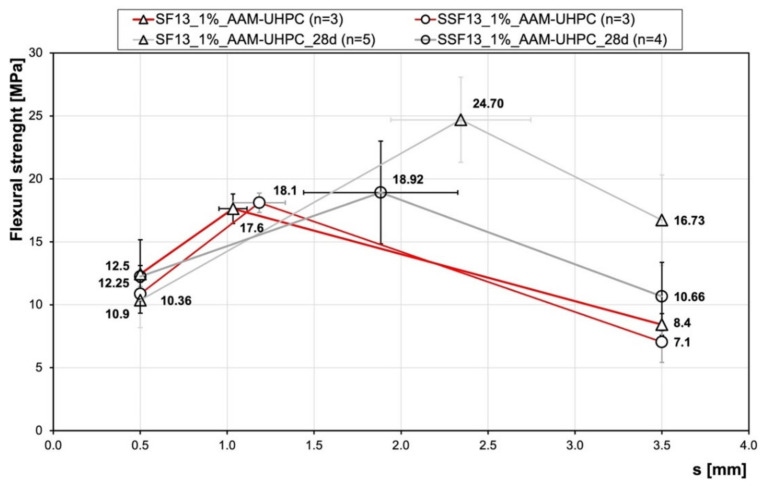
Flexural strength of AAM-UHPC after 7 (red curve) and 28 days (grey curve). The number n of tests is given in the legend.

Figure 3 and Figure 4 show the average flexural strengths of the test samples with steel and stainless-steel fibres (SFs and SSFs) for a fibre content of 1.0 vol% after 7 days and 28 days (Figure 4). The results are represented by the three characteristic values described above according to the dAfStb-guideline [18], which reflects the characteristic ranges and considers the standard deviations. The outlier samples of the strongly scattering measured values or the untypical curves were taken out.

Table 3 lists all of the values determined for the two binder variations and the fibre types by a fibre content of 1.0 vol% after 7 days. Summarized are the mean values of the maximum force and the resulting flexural strengths. Moreover, the performance factor was used for a direct comparison of the average characteristic values in the service range and fracture range. Likewise, the dissipated energy over the flexural course up to a deflection of 5 mm for comparison is given.

In the case of the OPC-UHPC, the specimens with the SF had a higher maximum flexural strength compared to the specimens containing the SSF. In contrast, the dissipated energy of the specimens with the SSF was higher compared to the specimens containing the SF. Furthermore, the SSF led to a higher displacement of the sample at the maximum flexural strength.

In the case of the AAM-UHPC, the maximum flexural strength was similar for both fibre types and in general was somewhat higher compared to the OPC-UHPC samples. Moreover, the dissipated energy was higher compared to the OPC-UHPC samples, so higher flexural strengths at larger deflections were obtained for the AAM-UHPC.

The results of the flexural strength of the AAM-UHPC after 28 days show a difference between the modes of action of the two types of fibres. Compared to the SSF, the specimens with the SF showed a maximum flexural strength that was about 23 percent higher (Table 4). In general, significantly higher flexural strength was obtained by the SF after 28 days. In addition, the maximum flexural strength at 28 days was obtained after late deflection, with the specimens with the SSF showing the largest difference at a displacement of about 2.4 mm compared to a displacement of about 1.0 mm after 7 days. This is consistent with the dissipated energy values after 28 days, which are generally higher than the values after 7 days. The SF samples were able to dissipate significantly more energy than the samples containing the SSF.

**Figure 5:**
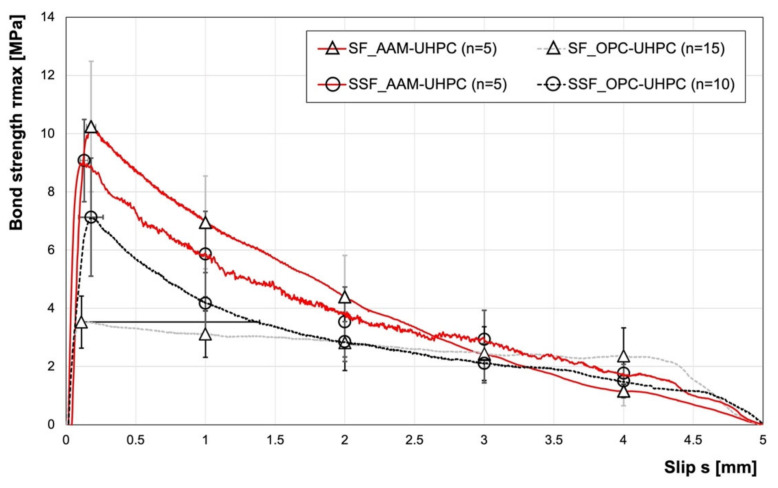
Fibre pullout of different fibres, as matrix AAM-UHPC and OPC-UHPC were used. The number n of tests is given in the legend.

The fibre-pullout tests showed a strong influence of the UHPC matrix on the bond between the fibre types and the concrete matrix. The average curves of the two fibre types (SFs and SSFs) from the AAM-UHPC and OPC-UHPC are shown in Figure 5. The indicated bond stresses were based on one fibre drawn from a specimen with five fibres. The bond stresses shown refer to the simultaneous extraction of five fibres from one specimen. The results showed that, in general, there was a higher bond strength between the fibres and the AAM-UHPC compared to the OPC-UHPC matrix. Considering the standard deviation, both fibre types were in a similar range. The SF showed the highest maximum bond stress with ~10 MPa, while the SSF had a maximum bond stress of ~9 MPa. The bond between the fibre and the OPC-UHPC matrix was lower compared to the AAM-UHPC and, moreover, showed a significant difference between fibre types, with maximum bond stresses of ~7 MPa and ~4 MPa for the SSF and SF, respectively (Table 5). The bond behaviour between the SF and the OPC matrix continued to have high maximum bond stresses via the frictional bond, so the maximum bond stress was reached at a higher slip (the standard deviation up to about 1.4 mm). This behaviour was different for the SFs and the AAM matrix. In this case, the maximum bond stress was reached after a slip of approx. 0.18 mm, and then, the curve decreased over the pullout.

**Figure 6:**
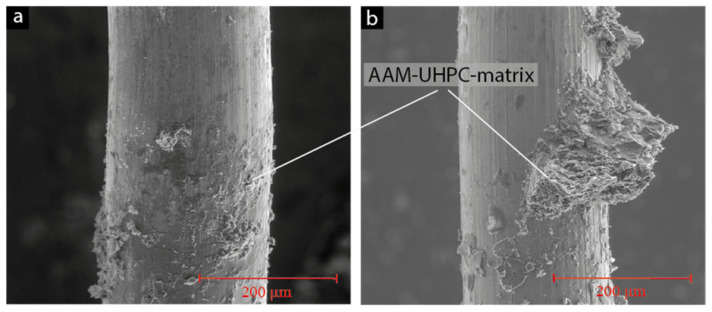
Microstructure on fibres in SEM, SF (**a**) and SSF (**b**) in secondary electron mode images.

The fibres were studied in a scanning electron microscope (SEM) directly after pullout experiments (Figure 6). In general, for both metal surface types, bigger parts of the AAM matrix, some measuring more than 100 µm, adhered to the metal fibres. The uppermost previously embedded parts are shown. Large parts of the surface were covered with the residual AAM matrix, which is why it might be assumed that cohesion failure within the AAM matrix occurred. 

**Figure 7:**
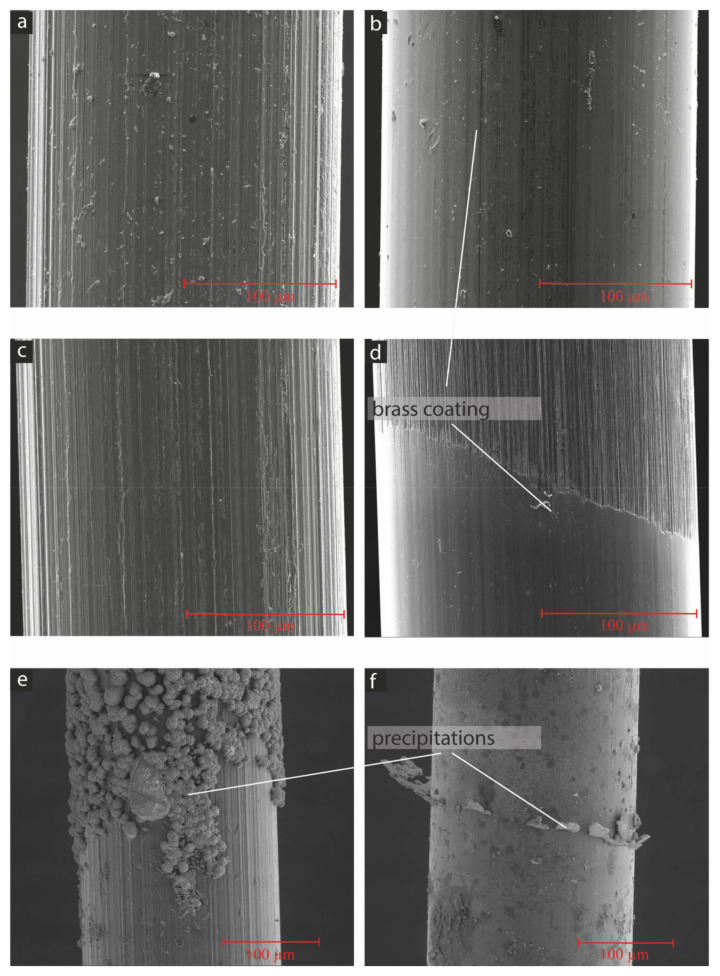
Microstructure on fibres in SEM, SSF (**a**,**c**,**e**), and SF (**b**,**d**,**f**) in secondary electron mode images, untreated (**a**,**b**), after 24 h in waterglass solution (**c**,**d**) and after 12 h in artificial pore solution (**e**,**f**).

**Figure 8:**
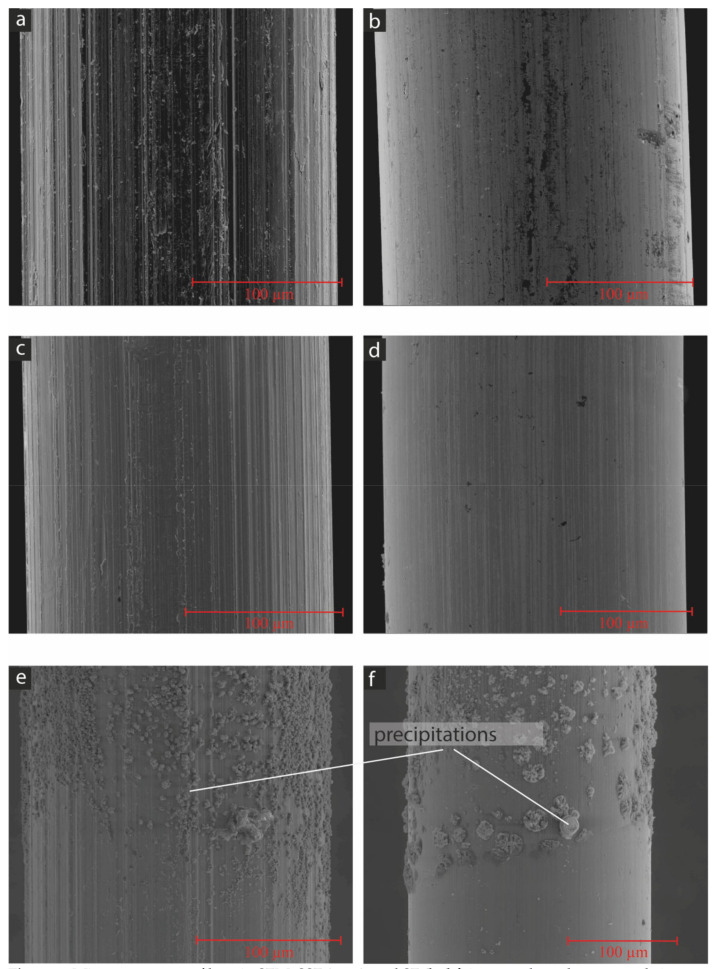
Microstructure on fibres in SEM, SSF (**a**,**c**,**e**), and SF (**b**,**d**,**f**) in secondary electron mode images, untreated (**a**,**b**), after 6 h in waterglass solution (**c**,**d**) and after 6 h in artificial pore solution (**e**,**f**).

To unravel the influence of the higher-alkaline environment in the case of the AAM-UHPC, some fibres (the SF and the SSF) were stored in the potassium waterglass solution used in mixtures M2–M4 (K57, see Table 1) compared to storage in an artificial pore solution [19] for different time periods. After 24 h, the brass coating of the steel fibres (SFs) was already dissolved after storage in the waterglass solution (Figure 7d, upper part). The resulting steel surface exhibited a higher roughness, which was comparable to the roughness of the SSF (Figure 7a,d). The surface of the SSF did not change after storage in the waterglass solution. Storing both fibre types in the artificial pore solution primarily showed precipitates, which might be calcium hydroxide, C-S-H phases and calcium-sulphate crystals. Anyhow, a dissolution of the brass coating on the SF and a change in the surface roughness cannot be recognized for both fibre types (Figure 7e,f). For a shorter time period of 6 h (Figure 8d), the dissolution of the brass coating only occurred for the waterglass solution, but not completely, as could be observed for the samples stored in the waterglass solution for 24 h. Less precipitations were observed on the fibres stored in the artificial pore solution (Figure 8e,f). 

## 4. Discussion

The modification of the mix design led to a higher compressive strength and an increase in the setting time. In particular, the setting time is quite important. While the setting time for M1 was about half an hour, it was increased to about two hours.

Already, small differences in the chemical composition of the ground-granulated, blast-furnace slag might lead to a change in the setting time [20]. Thus, the mix designs M2–M4 are much more robust concerning changes in the raw-material charges. The use of the waterglass with a modulus of 1 instead of the mix of alkali-hydroxides with alkali-waterglass (modulus 3.9) changed the rheology [21] towards higher slump values. Thus, the water/binder-ratio, and with this, the total amount of activator could be reduced leading to a further increase in compressive strength. This increase is due to the reduced capillary porosity, which in turn will probably lead to a further increase in durability. In the last optimization step from M3 to M4, the type of silica fume was changed. Generally, f-silica is much less cost intensive due to impurities concerning the SiO_2_ content. In particular, the residual-carbon content (LOI, see Table 1) is much higher, which is generally an indicator of inferior quality. Anyhow, in this UHPC based on AAM, the higher carbon content does not show any negative impact on fresh and hardened concrete properties.

The flexural strength for the AAM-UHPC with a content of 1 vol% of fibres, for both steel and stainless-steel fibres (M2 plus fibre), were higher compared to the OPC-UHPC. After 7 days, the flexural strength of the SSF sample of the OPC-UHPC was significantly lower compared to the OPC-UHPC with the SF and both mixtures of the AAM-UHPC (the SF and the SSF). This gets more obvious by not only comparing the maximum flexural strength but also the performance factor and the total dissipated energy (Table 3).

The bond behaviour of both fibre types, stainless-steel (SSF) and brass-coated steel (SF), which was determined by straight multiple-fibre pullout tests, revealed a good bond behaviour to the AAM-UHPC matrix. In comparison to the UHPC based on the OPC [4], higher maximum bond stresses were determined by both fibre types in the adhesive and frictional bond, resulting in a higher dissipated energy and utilization rate. The difference between the PC-based and the AAM-based systems is comparable to results reported elsewhere [22,23]. The bond stress–slip curve of the SF differed depending on the UHPC matrix, while the OPC-UHPC showed a high friction bond and the friction bond of the AAM-UHPC decreased after maximum bond stress.

Compared to the OPC-UHPC [9], the bond with the metallic surface seemed strengthened for the AAM-UHPC. The surface of the SF changed due to the alkaline environment; the brass coating etched off. This enhanced the surface roughness and affected enhanced bonding to the AAM-UHPC matrix. Compared to fibres pulled out of the OPC-UHPC, those pulled out of the AAM-UHPC seemed to show a higher roughness anyway [9]. The smaller difference for the SF and the SSF in the AAM-UHPC can be explained by a comparable roughness, which resulted from etching of the brass coating of the SF. Further investigations are needed to determine how the highly alkaline environment of the fresh AAM-UHPC attacks the surface of the steel fibres and enables a better bond, or whether C-A-S-H and N-A-S-H phases [24] can produce a better bond to metal surfaces compared to C-S-H phases. Although, the AAM-UHPC is characterized by a very low w/b ratio and thus a low capillary porosity and a high durability, so the steel corrosion in the AAM would be different from the corrosion processes in OPC-based systems [25]. Thus, durability aspects need to be investigated in future. 

## 5. Conclusions

The AAM-UHPC was optimized by changing the waterglass solution towards a lower modulus; thus, only one activator component needed to be added. The compressive strength, setting time, and slump flow were improved by that optimization step. As the bending strength of the UHPC without reinforcement is rather low, fibres, mainly steel fibres, were added to reduce this disadvantage. It was shown that the bond of the steel fibres to the AAM-UHPC was comparable to the OPC-UHPC and even better. Not only is the maximum value of the bending strength of major interest, but so is the performance factor, which gives information about the post-cracking behaviour. This performance factor showed a difference towards the different fibre types used; for the different binder types, the difference was even bigger. The differences in the fibre types (SF, SSF) were low compared to the difference in the fibre pullout, especially compared to the pullout tests of the fibres and the OPC-UHPC. Experiments on the fibres stored in a waterglass and an artificial pore solution indicated that the improved bond of the steel fibres to the AAM resulted from the slight dissolution of the surface, which increased the topography and therefore the friction during pullout.

## Figures and Tables

**Table 1:** Properties of raw materials.

		Slag	Metakaolin	Silica Fume	F-Silica Fume
SiO_2_	(wt%)	35.80	52.67	96.57	88.77
Al_2_O_3_	(wt%)	11.70	42.91	0.06	0.76
Fe_2_O_3_	(wt%)	0.37	0.33	0.06	1.31
CaO	(wt%)	43.80	0.03	0.51	0.20
MgO	(wt%)	5.80	0.07	0.25	0.55
MnO	(wt%)	0.30	0.01	0.02	0.13
K_2_O	(wt%)	0.40	0.53	0.73	2.06
Na_2_O	(wt%)	0.20	0.04	0.16	0.24
SO_3_	(wt%)	0.17	0.04	0.12	0.76
TiO_2_	(wt%)	1.08	0.03	0.01	0.04
LOI	(wt%)	0.27	3.25	1.41	4.73
spec. surf.	(cm^2^/g)	3900	91,100	218,000	193,200
d10	(µm)	1.2	1.5	0.3	0.2
d50	(µm)	10.4	5.2	0.6	0.4
d90	(µm)	31.7	20.8	1.9	9.8

**Table 2:** Mix composition for AAM-UHPC and OPC-UHPC formulation as a reference (ref: Wiemer et al. 2020 [9].)

		M1	M2	M3	M4	UHPC^ref^ (OPC)
Quartz sand	(wt%)	50	50	50	50	41.2
Quartz powder	(wt%)	8	8	8	8	8.5
Blast furnace slag (BFS)	(wt%)	27	27	27	27	
CEM I 52.5R SR/NA	(wt%)	-	-	-	-	34
Silica fume 1 (SF1)	(wt%)	15	15	15	-	7.2
Silica fume 2 (SF2)	(wt%)	0	-	-	15	-
Waterglass modulus	(SiO_2_:K_2_O)	3.9 ^#^	1 ^+^	1 ^+^	1 ^+^	-
KOH 10 molar		✓	-	-	-	-
KOH (10M)/K_2_SiO_3_ (3.9)	(wt./wt.)	1.5	-	-	-	-
Superplasticizer (PCE)	(wt% bwoc)	-	-	-	-	1.3
w/b ratio *	(wt./wt.)	0.250	0.230	0.175	0.175	0.21
Comp. strength (7d)	(MPa)	108.0	162.9	188.4	190.9	138.2
Comp. strength (28d)	(MPa)	157.5	194.2	211.8	212.8	176.7
Slump flow	(mm)	220	340	300	240	280
Setting time	(min)	29	116	106	100	560

* excluding solid content of activator solution. ^#^ solid content of 40%. ^+^ solid content of 53%.

**Table 3:** Comparison of the flexural strength values of SSF and SF as a function of concrete composition.

	SF13_1%_OPC-UHPC			SSF13_1%_OPC-UHPC		
	δ_L1_ = 0.5 mm	δ_L2_ = 3.5 mm	δ_L(max)_	δ_L1_ = 0.5 mm	δ_L2_ = 3.5 mm	δ_L(max)_
mean forces [kN]	7.59	1.55	10.59	5.26	3.64	8.93
standard deviation F [kN]	0.90	0.28	0.98	2.06	1.70	1.30
standard deviation s [mm]	0.00	0.00	0.26	0.00	0.00	0.54
average f_cflm_ [MPa]	11.86	2.41	16.55	8.21	5.69	13.95
f_cflk_ [MPa]	6.05	1.23	8.44	4.19	2.90	7.11
performance factor	L 6.05/1.23			L 4.19/2.90		
dissipated energy [Nm]	22.03 ± 3.2			24.12 ± 2.9		
		
	SF13_1%_AAM-UHPC			SSF13_1%_AAM-UHPC		
	δ_L1_ = 0.5 mm	δ_L2_ = 3.5 mm	δ_L(max)_	δ_L1_ = 0.5 mm	δ_L2_ = 3.5 mm	δ_L(max)_
mean forces [kN]	7.97	5.40	11.28	6.96	4.52	11.59
standard deviation F [kN]	0.42	0.55	0.75	0.37	1.04	0.49
standard deviation s [mm]	0.00	0.00	0.08	0.00	0.00	0.15
average f_cflm_ [MPa]	12.45	8.43	17.63	10.87	7.05	18.11
f_cflk_ [MPa]	6.35	4.30	8.99	5.54	3.60	9.24
performance factor	L 6.35/4.30			L 5.54/3.60		
dissipated energy [Nm]	34.04 ± 2.9			31.21 ± 2.9		

**Table 4:** Comparison of the flexural strength values after 28 days of SSF and SF as a function of concrete composition.

	SF13_1%_AAM UHPC_28d			SSF13_1%_AAM-UHPC_28d		
	δ_L1_ = 0.5 mm	δ_L2_ = 3.5 mm	δ_L(max)_	δ_L1_ = 0.5 mm	δ_L2_ = 3.5 mm	δ_L(max)_
mean forces [kN]	6.63	10.70	15.81	7.84	6.82	12.11
standard deviation F [kN]	1.39	2.29	2.17	1.87	1.74	2.61
standard deviation s [mm]	0.00	0.00	0.40	0.00	0.00	0.44
average f_cflm_ [MPa]	10.36	16.73	24.70	12.25	10.66	18.92
f_cflk_ [MPa]	5.28	8.53	12.59	6.25	5.44	9.65
performance factor	L 5.28/8.53			L 6.25/5.44		
dissipated energy [Nm]	52.93 ± 8.3			38.71 ± 7.6		

**Table 5:** Results of the fibre-pullout tests for the different fibres, as matrix AAM-UHPC and OPC-UHPC were used.

	F_max_	F_max_/ Fibre	F_y_	D_f_	sF_max_	τ_Max_	σ_f,Max_/ Fibre	U_f_	W_p_
	N	N	MPa	mm	mm	MPa	MPa	%	Nmm
SF_AAM	160.9	32.2	2000	0.20	0.18	10.3	1024	51.2	60.7
SF_OPC	69.2	13.8	2000	0.25	0.11	3.5	282	14.1	49.3
SSF_AAM	142.6	28.5	1980	0.20	0.13	9.1	907	45.8	57.3
SSF_OPC	112	22.4	1980	0.20	0.18	7.1	713	36.0	43.9

F_max_: maximum pullout load, F_max_/fibre: maximum pullout load per fibre, F_y_: tensile strength of the fibre, sF_max_: fibre-pullout load at maximum pullout load, D_f_: fibre diameter, τ_max_: maximum bond stress, σ_f,max_/fibre: maximum reached fibre tensile stress, U_f_: capacity utilization of the fibre; W_p_: pullout work.

## Data Availability

Not applicable.
